# A comparative study of the gut microbiome in Indian children with type 1 diabetes and healthy controls

**DOI:** 10.1111/1753-0407.13438

**Published:** 2023-06-28

**Authors:** Vaishali Tamahane, Shivang Bhanushali, Nikhil Shah, Abhishek Gupta, Vaman Khadilkar, Ketan Gondhalekar, Anuradha Khadilkar, Yogesh Shouche

**Affiliations:** ^1^ Hirabai Cowasji Jehangir Medical Research Institute Jehangir Hospital Pune India; ^2^ School of Health Sciences‐Savitribai Phule Pune University Pune India; ^3^ National Centre for Microbial Resource—National Centre for Cell Science Pune India

**Keywords:** glycemic control, gut microbiome, Indian children, short chain fatty acids, type 1 diabetes

## Abstract

**Introduction:**

Type 1 diabetes mellitus (T1DM) occurs in genetically susceptible individuals due to certain environmental triggers causing destruction of insulin secreting beta cells. One of the environmental triggers studied recently in the pathogenesis and progression of T1DM is the role of gut microbiome.

**Objectives:**

(1) To compare the gut microbiome profile of T1DM children with healthy age, gender, and body mass index (BMI) matched controls. (2) To assess the relationship of abundance of genera with glycemic control in children with T1DM.

**Methods:**

Cross‐sectional, case–control study. Sixty‐eight children with T1DM and 61 age‐, gender‐, and BMI‐matched healthy controls were enrolled. QIAamp Fast DNA Stool Mini kit protocol and reagents were used for DNA isolation and Miseq sequencing platform for targeted gene sequencing.

**Results:**

Alpha and beta diversity analysis showed no significant differences in the abundance of microbes between the groups. At phylum level, Firmicutes was the dominant phylum followed by Actinobacteria and Bacteroidota in both groups. Analysis of microbiome at the genera level showed that percentage abundance for *Parasutterella* was higher in children with T1DM as compared to the healthy group (*p* < .05). A linear regression analysis showed that increase in abundance of *Haemophilus* (adjusted *R* ^2^ = −1.481 *p* < .007) was associated with a significant decrease in glycated hemoglobin (HbA1c) concentrations (*p* < .05).

**Conclusion:**

Our comparative study of gut microbiome profile showed significant differences in the taxonomial composition between Indian children with T1DM and healthy controls. Short chain fatty acid producers may play an important role in glycemic control.

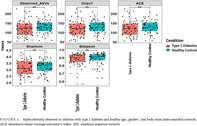

## Graphical abstract

Highlights

- To best of our knowledge, this is the first study conducted to compare the gut microbiome of Indian children with type 1 diabetes mellitus (T1DM) and healthy controls.
- There were significant differences observed in the gut microbiome profile at the genera level of children with T1DM as compared to controls, thus indicating a distinct gut microbial signature in children with type 1 diabetes.
- Short chain fatty acid producers may play an important role as significant predictor of glycemic control in Indian children with T1DM.

## 1 INTRODUCTION

The gut microbiome of humans is made up of several different types of microbes that reside in the gastrointestinal tract. This group of bacteria, archaea, and eukaryote living in the human gut interact and coevolve with the host system and are collectively called the gut microbiome.^1^ The human gut contains more than 10^14^ bacteria. The most dominant genera found in the healthy human gut include *Bacteroides*, *Clostridium*, *Fusobacterium*, *Eubacterium*, *Ruminococcus*, *Peptococcus*, *Peptostreptococcus*, and *Bifidobacterium*.^2^ The other genera like *Escherichia* and *Lactobacillus* are also present in the gut but with a lower abundance.^3^

Any disturbance such as a change in dietary pattern induces a change in the composition of the gut microbiome leading to a condition labeled as dysbiosis.^4^ Dysbiosis has been implicated in the pathogenesis of several chronic disorders such as type 1 diabetes mellitus (T1DM), inflammatory bowel disorder, irritable bowel syndrome, and obesity. T1DM is an autoimmune disorder that occurs in genetically susceptible individuals due to certain environmental triggers resulting in the destruction of insulin‐secreting pancreatic beta cells.^5^ One of the environmental triggers postulated and studied in recent years is the role of the gut microbiome in the pathogenesis of T1DM.

The association between microbiome maturation and immune system development with parallel production of autoantibodies in T1DM is not fully understood. It is not known whether this interplay is temporary or is a causative factor for T1DM in young ones. Gérard et al. proposed several molecular mechanisms like modulation of incretin secretion, short chain fatty acid (SCFA) production, bile acid transformation, and regulation of adipose tissue inflammation and function, with the help of which the gut microbiota gets modulated and interferes with the glycemic control of the host.^6^ Studies have reported that the reduced abundance of SCFA producing bacteria is associated with signs of beta cells auto‐immunity.^7^

As per the International Diabetes Federation 2021 atlas, the highest number of children with T1DM (aged 0–19 years) reside in India. Further, several studies also report that the incidence and prevalence of T1DM is rapidly increasing.^8^ The majority of the studies done on the gut microbiome in children with T1DM have been conducted in developed countries. Given that in Indian children with T1DM, the gut microbiome might be considerably different from that reported in the populations from the developed countries, especially with different genetic risk scores, different diet patterns, and an increased prevalence of intercurrent gut infections, we performed this study with the following objectives: (1) to compare the gut microbiome profile of Indian children with T1DM with healthy age‐, gender‐, and body mass index (BMI)‐ matched controls; and (2) to assess the relationship of abundance of genera (gut microbiome) with glycemic control as judged by the glycated hemoglobin (HbA1c) in Indian children with T1DM.

## 2 METHODS

### 2.1 Study design

Cross‐sectional, case–control study.

### 2.2 Study subjects

**FIGURE 1:**
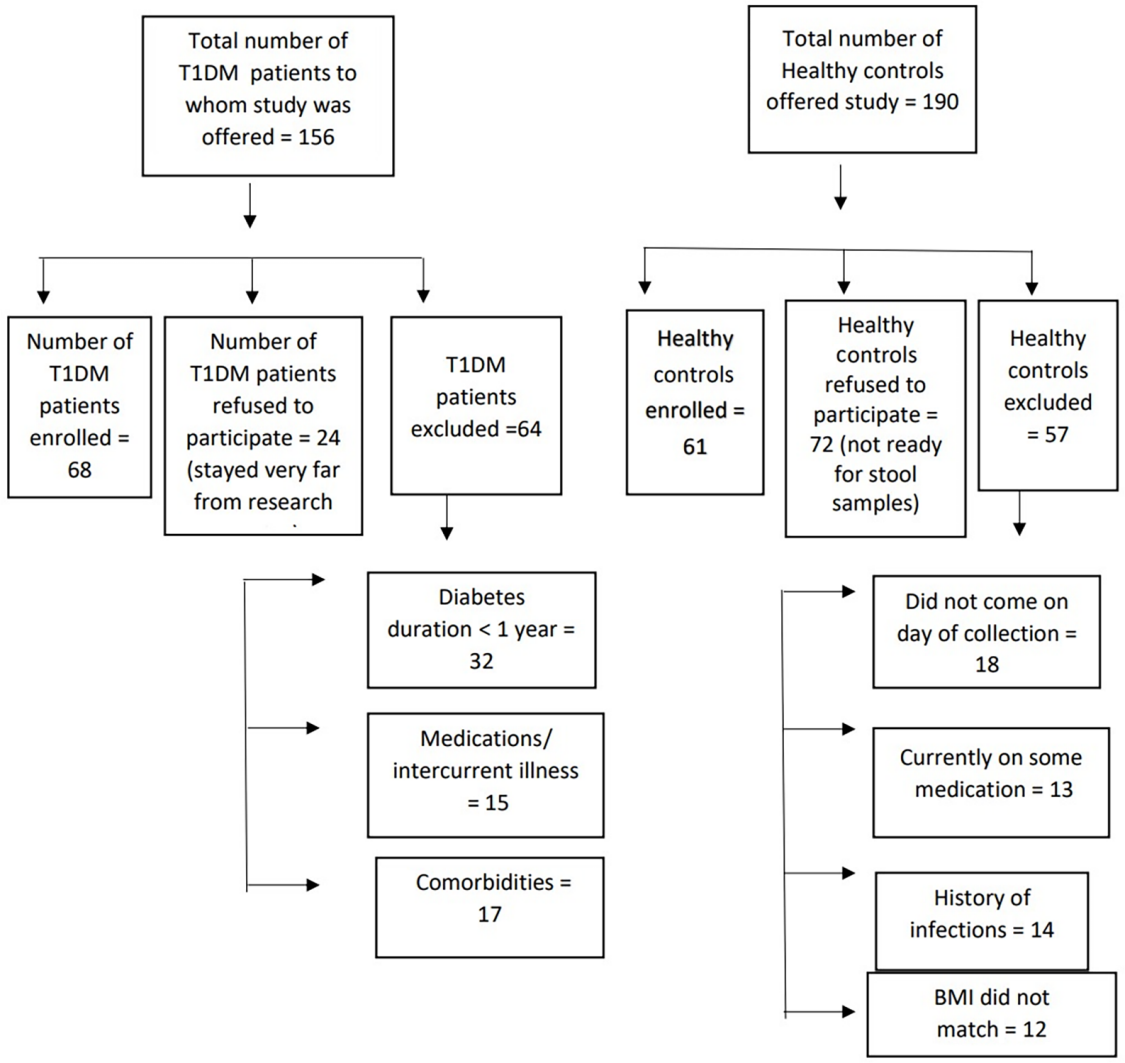
Flow chart for subject enrollment. BMI, body mass index; T1DM, type 1 diabetes mellitus.

Children with T1DM attending a tertiary level care pediatric endocrine clinic, in Pune, Maharashtra, India, during the study period were offered the study (October 2020 to March 2021). Age‐ and gender‐matched healthy children were enrolled from the communities of children with T1DM. The healthy control group was enrolled from a similar sociodemographic environment. The participant flow is illustrated in Figure 1.

#### 2.2.1 Inclusion criteria

Children with T1DM with disease duration for at least a period of 1 year and whose parents gave written informed consent and children gave assent.^9^

#### 2.2.2 Healthy controls

Age‐, gender‐, and BMI‐matched children from similar backgrounds as the children with T1DM.

#### 2.2.3 Exclusion criteria

Children with T1DM or healthy controls on antibiotic therapy in the last 3 months or with history of diarrhea in last 15 days. Further, presence of any other comorbidities/chronic disease (gluten sensitivity, celiac disease, uncontrolled autoimmune thyroiditis) or on any medication altering renal function or lipid concentrations.

An ethics approval for conducting the study was obtained from the Institutional Ethics Committee (Jehangir Clinical Development Centre dated 24 September 2020). All parents signed a written informed consent and the children signed an assent before any study procedures were performed. The study was conducted during October 2020–March 2021.

A total of 68 children with T1DM and 61 age‐ and gender‐matched healthy controls were enrolled. A post hoc power calculation (G‐power 3.1 version), at a significance level of 0.05 level for a sample size of 68, power of 0.8 was obtained.

### 2.3 Clinical history and anthropometric parameters

Medical history (including mode of delivery, ie, vaginal or cesarean) of all the children with T1DM was recorded using a questionnaire and verified from medical records. Clinical examination was performed by a pediatric endocrinologist to ascertain inclusion into the study. History was also recorded from healthy controls to rule out diarrhea, intake of antibiotics, etc. Height (stadiometer) and weight (digital scale) were recorded using standard protocols and BMI was calculated (weight in kilograms by height in meters square). *Z*‐scores for height, weight, and BMI were calculated using Indian references.^10^ To assess the glycemic control, HbA1c was measured in children with T1DM using a d10 HbA1c analyzer.

### 2.4 DNA extraction and 16srRNA sequencing and bioinformatics analysis

#### 2.4.1 Fecal sample collection

The study participants were requested to come to the center for the first morning fecal sample. Stool samples were collected in prelabeled, sterile containers and were frozen immediately at −80°C until further analysis.

#### 2.4.2 Fecal DNA isolation

QIAamp Fast DNA Stool Mini kit protocol and reagents were used for DNA isolation.

#### 2.4.3 16SrRNA sequencing method

Miseq sequencing platform was used for targeted gene sequencing. Total community DNA was extracted from each fecal sample using QIAamp DNA Stool Mini kit (Qiagen, Madison, WI, USA) as per the manufacturer's protocol. Polymerase chain reaction (PCR) amplification and sequencing of resulting amplicons was performed. The concentration of extracted DNA was measured using Nanodrop‐1000. DNA concentration was normalized to 100 ng/μL and used as a template for amplification of 16SrRNA gene. PCR was set up in 25 μL reaction using 1x PCR master mix (Takara LA Taq) and with 1.5 mM MgCl_2_, 30 ng DNA as a template and 10 picomole each primer, 16SrRNA V4 region specific bacterial universal primers. The following conditions were used for PCR: initial denaturation at 95°C for 3 min, followed by 25 cycles of 95°C for 30 s, 55°C for 30 s, and 72°C for 30 s with final extension at 72°C for 5 min. PCR products were purified using 1X AMPure XP Reagent (Beckman Coulter) and were pooled together at 4 nM concentration. The resulting pooled libraries were sequenced on Illumina MiSeq platform with 2 × 250 bp paired‐end sequencing according to the Illumina 16S Metagenomic Sequencing Library Preparation.^11^ The obtained paired‐end raw reads were checked for quality using FastQC^12^ followed by analysis using DADA2 package version 1.6.0 (Callahan et al.,^13^) in R 3.6.0. The low‐quality reads and primers were removed using filtertrim function (trimLeft = c(19, 20), truncLen = c(), maxN = 0, maxEE = c(2,2), truncQ = 2, and rm.phix = TRUE). The chimeric reads were removed using remove Bimera Denovo function of DADA2. Silva Database (silva_nr99_v138.1_train_set.fa.gz) was used for the taxonomic assignment of the amplicon sequence variants (link silva). Core genera were identified using microbiome R package (Lahti and Shetty^14^). Wilcoxon test was used to assess the differences in alpha diversity parameters between the study groups using ggpubr R package (Kassambara and Kassambara^15^).

#### 2.4.4 Diversity analysis

Alpha diversity for a single community was measured by using several estimators like Chao1 and abundance‐based coverage estimators (ACE). Alpha diversity measures the number and distribution of taxa expected in a single population. Abundance‐based coverage estimator, estimates the relative abundance of undetected species using information from rare species group. Chao1 is a non‐parametric index for estimating number of rare species in the community and gives information on the number of missing species. Shannon's diversity index is defined as a heterogeneity measure which considers the degree of evenness in species abundance.^16^ It is used to characterize the species diversity in a community. Simpson's Diversity Index is defined as a measure of diversity that considers the number of species present, as well as the relative abundance of each species.^17^ Beta diversity is defined as a measure of similarity and dissimilarity with respect to the number of taxa shared between two different communities.^18^

### 2.5 Statistical tests

All statistical analyses were performed using a statistical software for social sciences (IBM SPSS‐26). Non‐parametric tests like Mann Whitney *U* test were used to estimate the abundance of genera (gut microbiome) profile in both groups. Spearman's rho was used to assess the correlation between the variables. Linear regression analysis was performed to assess the associations of HbA1c in children with T1DM. The value of *p* < .05 was considered significant.

## 3 RESULTS

A total of 129 participants were enrolled (68 children with T1DM and 61 age, gender and BMI matched healthy controls). The mean age was 12.4 ± 3.6 years and 12.1 ± 2.9 years for children with T1DM and healthy controls respectively. The average HbA1c was 9.8 ± 1.8% and mean disease duration was 5.5 ± 3.0 years in children with T1DM. The anthropometric and clinical parameters of the participants are illustrated in Table 1; no significant differences were observed between the two groups (*p* > .1). Fifty‐six (82.3%) children with T1DM and 50 (82%) healthy controls were born through vaginal delivery, whereas, 12 (17.7%) children with T1DM and 11 (18%) healthy controls were delivered through cesarean section. There were no significant differences in the proportions of children with T1DM delivered through vaginal delivery or cesarean section as compared to the healthy controls (*p* > .1).

**TABLE 1:** Anthropometric parameters of children with type 1 diabetes and healthy age‐, gender‐, and body mass index‐matched controls.

Parameter (SI units)	Children with type 1 diabetes (*n* = 68)	Healthy children (*n* = 61)	*p* value
Age (years)	12.4 ± 3.6	12.1 ± 2.9	0.513
Height (m)	1.4 ± 0.1	1.4 ± 0.1	0.641
Height *Z*‐scores	−0.5 ± 1.1	−0.2 ± 1.7	0.208
Weight (kg)	39.3 ± 11.1	38.3 ± 12.1	0.647
Weight *Z*‐scores	−0.3 ± 0.9	−0.4 ± 1.4	0.655
Body mass index (kg/m^2^)	18.1 ± 3.4	17.4 ± 3.5	0.230
Body mass index *Z*‐scores	−0.1 ± 0.9	−0.4 ± 1.1	0.679
HbA1c (%)	9.8 ± 1.8	NA	‐
Disease duration (years)	5.5 ± 3.0	NA	‐

*Note*: All values are expressed in mean ± standard deviation.

Abbreviations: HbA1c, glycated hemoglobin; NA, not applicable.

**FIGURE 2:**
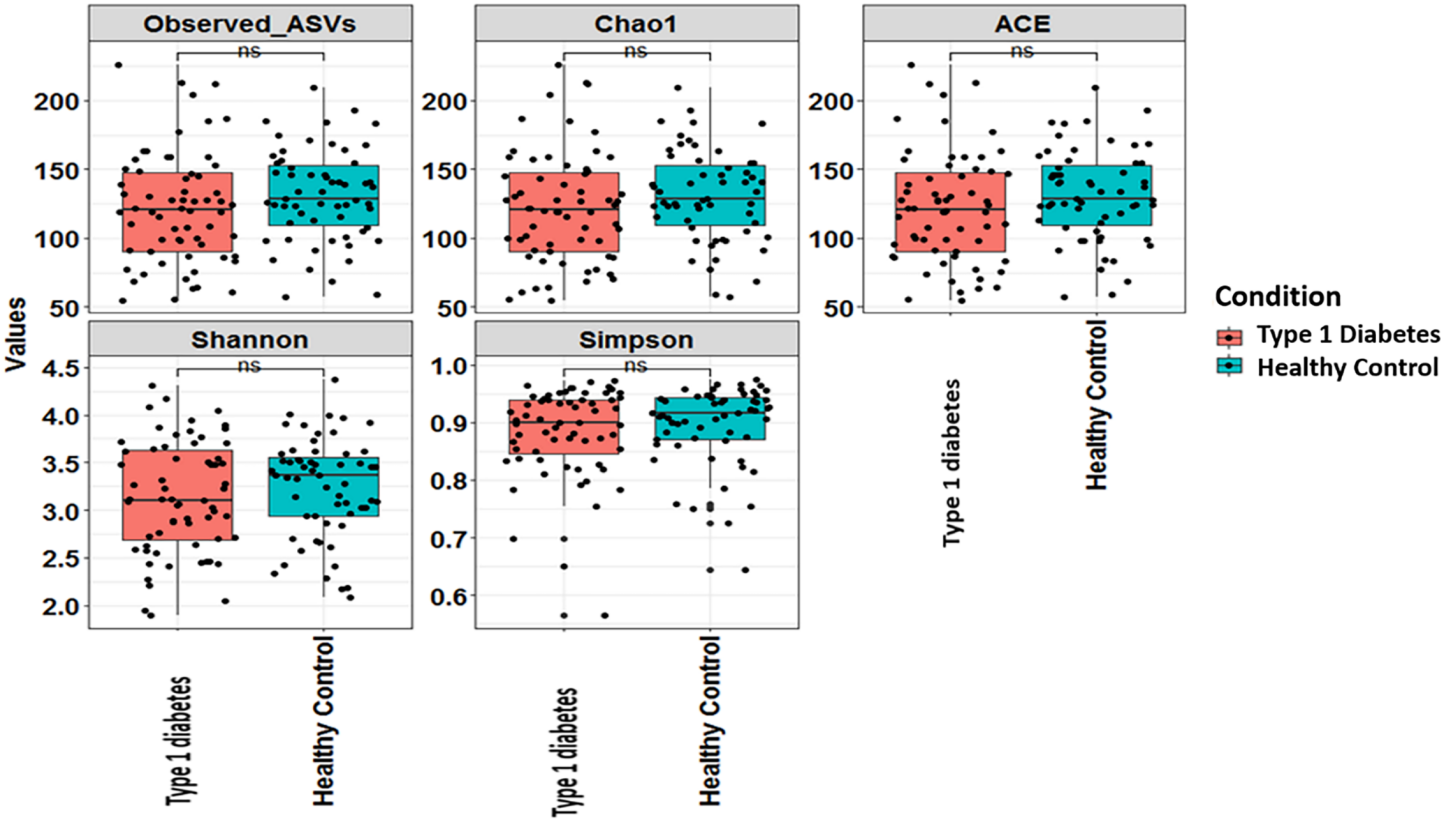
Alpha‐diversity observed in children with type 1 diabetes and healthy age‐, gender‐, and body mass index‐matched controls. ACE, abundance‐based coverage estimator''s index; ASV, amplicon sequence variants.

When alpha diversity analysis was performed, no significant differences (*p* > .1) were observed in the species richness, evenness, amplicon sequence variants (ASVs), Chao 1, abundance‐based coverage estimator's index (ACE index), Shannon's index, and Simpson's index indicating similar microbial diversity composition in both groups as illustrated in Figure 2 (*p* > .1).

**FIGURE 3:**
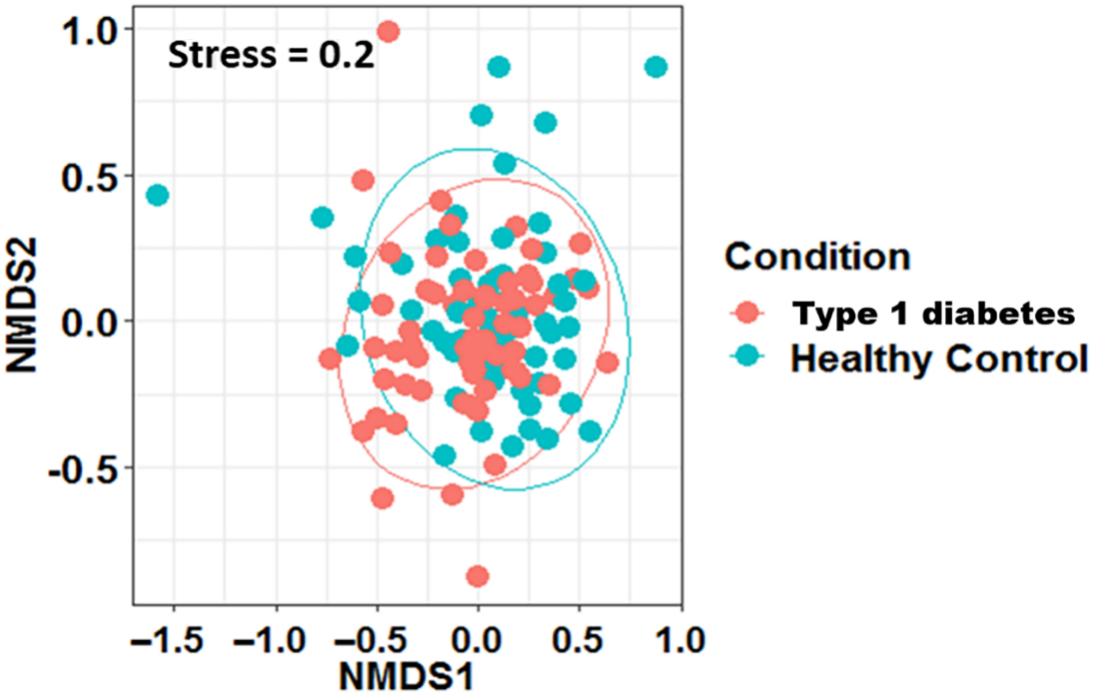
Nonmetric multidimensional scaling (NMDS) plot based on genera (Beta diversity) in children with type 1 diabetes and healthy age‐, gender‐, and body mass index‐matched controls.

On performing beta diversity analysis (Figure 3), at the genera level, there were no significant differences in the abundance of microbes in children with diabetes and healthy age‐, gender‐, and BMI‐matched controls. Nonmetric multidimensional scaling did not show any distinct clusters between the two groups with respect to age and gender, which was further confirmed by analysis of similarities (*p* > .1).

**FIGURE 4:**
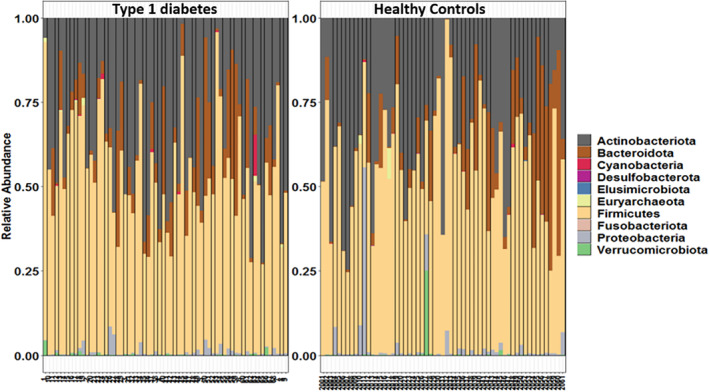
Phyla‐level distribution of gut microbiome in children with type 1 diabetes and healthy age‐, gender‐, and body mass index‐matched controls.

**FIGURE 5:**
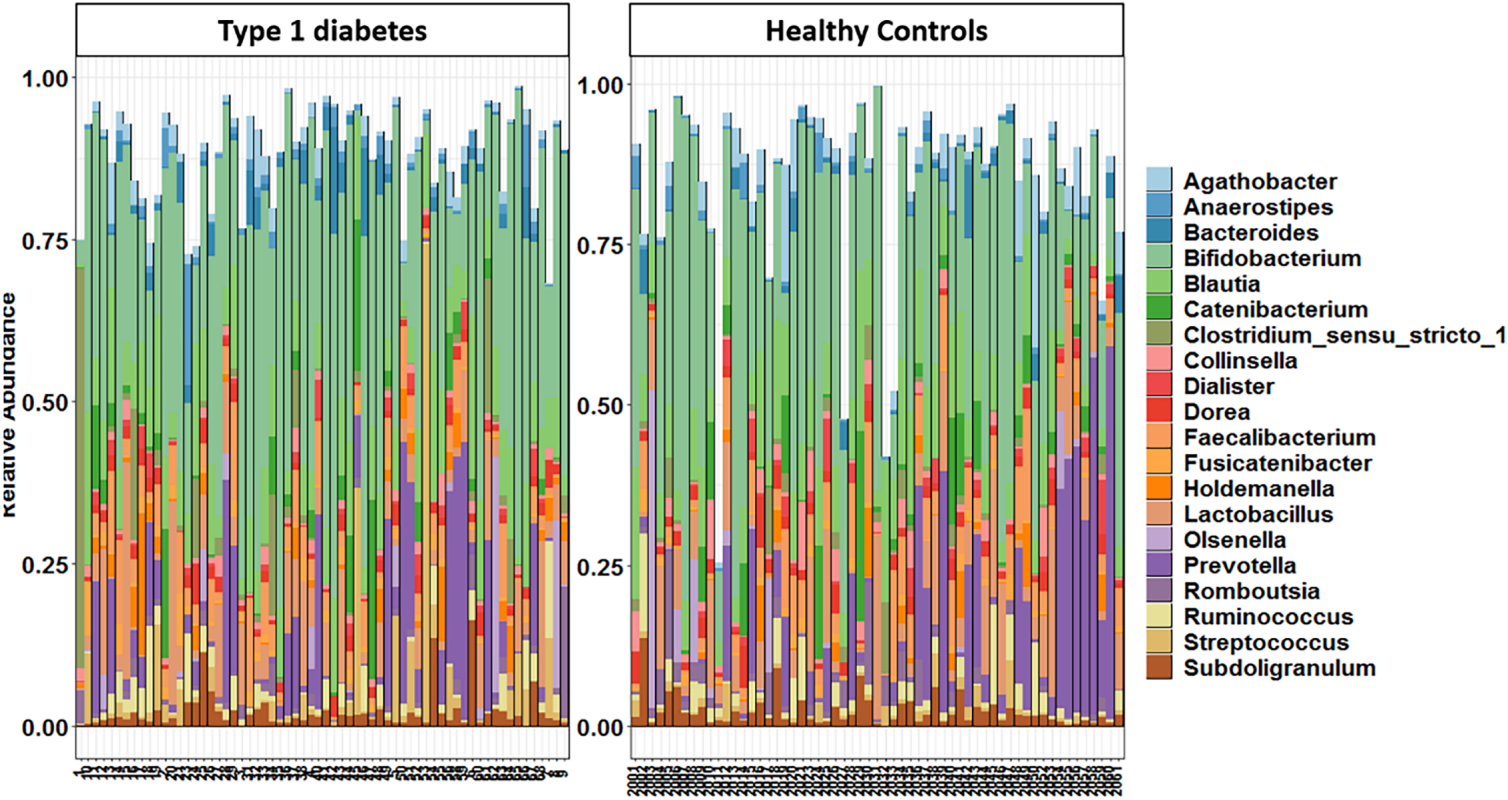
Genera‐level distribution of the gut microbiome in children with type 1 diabetes and healthy age‐, gender‐, and body mass index‐matched controls.

At the phylum level, it was observed that Firmicutes was the dominant phylum followed by Actinobacteria and Bacteroidota across both the study groups as illustrated in Figure 4. Actinobacteria and Proteobacteria were found to be abundant in both groups. Cyanobacteria was found to be abundant in T1DM group and Verrucomicrobiota was abundant in healthy controls. A comparative analysis of abundance of gut microbiome at genus level showed that percentage of abundance of reads for *Acinetobacter*, *CoriobacteriaceaeUCG003*, *Raoultibacter* was higher in the healthy group and abundance of *Parasutterella* was higher in children with T1DM as illustrated in Table 2. *Blautia* and *Prevotella* were the abundant genera found in both groups (Figure 5). *Bifidobacterium* was the only genus recorded as a core microbiome member with prevalence greater than 90% in samples in both groups.

**TABLE 2:** Comparative analysis of abundance of gut microbiome profile at genera level in children with type 1 diabetes and healthy age, gender and body mass index matched controls.

Genera (Reads of abundance)	Children with type 1 diabetes (*n* = 68)	Healthy children (*n* = 61)
Anaerostipes (%)	95.3	93.2
Acinetobacter (%)^a^	3.1	15.3
Parasutterella (%)^a^	28.1	11.9
CoriobacteriaceaeUCG003 (%)^a^	0.0	6.8
Raoultibacter (%)^a^	1.6	10.2
Butyricicoccus (%)	89.1	88.1
Haemophilus (%)	76.6	69.5
FamilyXIIIUCG001 (%)	17.2	25.4
Mogibacterium (%)	7.8	11.9

*Note*: All values corresponding to each genus in the above table represent proportion (%) present in children with type 1 diabetes and healthy children.

^a^ Represents values significant at *p* < 0.05.

The abundance of genera like *Haemophilus* and *Mogibacterium* showed a negative correlation with HbA1c, *r* ^2^ = −0.287 (*p* = .004) and *r* ^2^ = 0.260 (*p* = .049) respectively, whereas *Butyricicoccus* (*r* ^2^ = 0.373), *FamilyXIIIUCG001* (*r* ^2^ = 0.359) and *Fournierella* (*r* ^2^ = 0.321) showed a positive correlation with HbA1c in children with T1DM (*p* < .05) as illustrated in Table 3.

**TABLE 3:** Relationship of abundance of genera with glycated hemoglobin in children with type 1 diabetes mellitus.

Genera (Reads of abundance)	Spearman's correlation coefficient	*p*‐value
Butyricicoccus (%)^a^	0.373	0.004
Haemophilus (%)^a^	−0.287	0.029
FamilyXIIIUCG001 (%)^a^	0.359	0.006
Fournierella (%)^a^	0.321	0.014
Mogibacterium (%)^a^	−0.260	0.049

^a^ Represents statistically significant values at *p* < 0.05.

A linear regression analysis was performed to determine which of the genera in the gut microbiome showed an association with HbA1c in children with T1DM as shown in Table 4. The increase in abundance of *Haemophilus* (adjusted *R* ^2^ = −1.481 *p* < .05) was associated with a significant decrease in HbA1c concentrations (*p* < .05). The other genera like *Butyricicoccus*, *FamilyXIIIUCG001*, *Fournierella*, and *Mogibacterium* did not show a significant association with HbA1c (*p* > .05).

**TABLE 4:** Associations of glycated hemoglobin in children with type 1 diabetes mellitus using linear regression.

Genera (Reads of abundance)	Unstandardized coefficients B	SE	*p*‐value
Butyricicoccus (%)	1.285	0.723	0.081
Haemophilus (%)^a^	−1.481	0.528	0.007
FamilyXIIIUCG001 (%)	1.046	0.610	0.093
Fournierella (%)	0.750	0.750	0.339
Mogibacterium (%)	−0.745	−0.745	0.375

^a^ Represents statistically significant values at *p* < 0.05.

**FIGURE 6:**
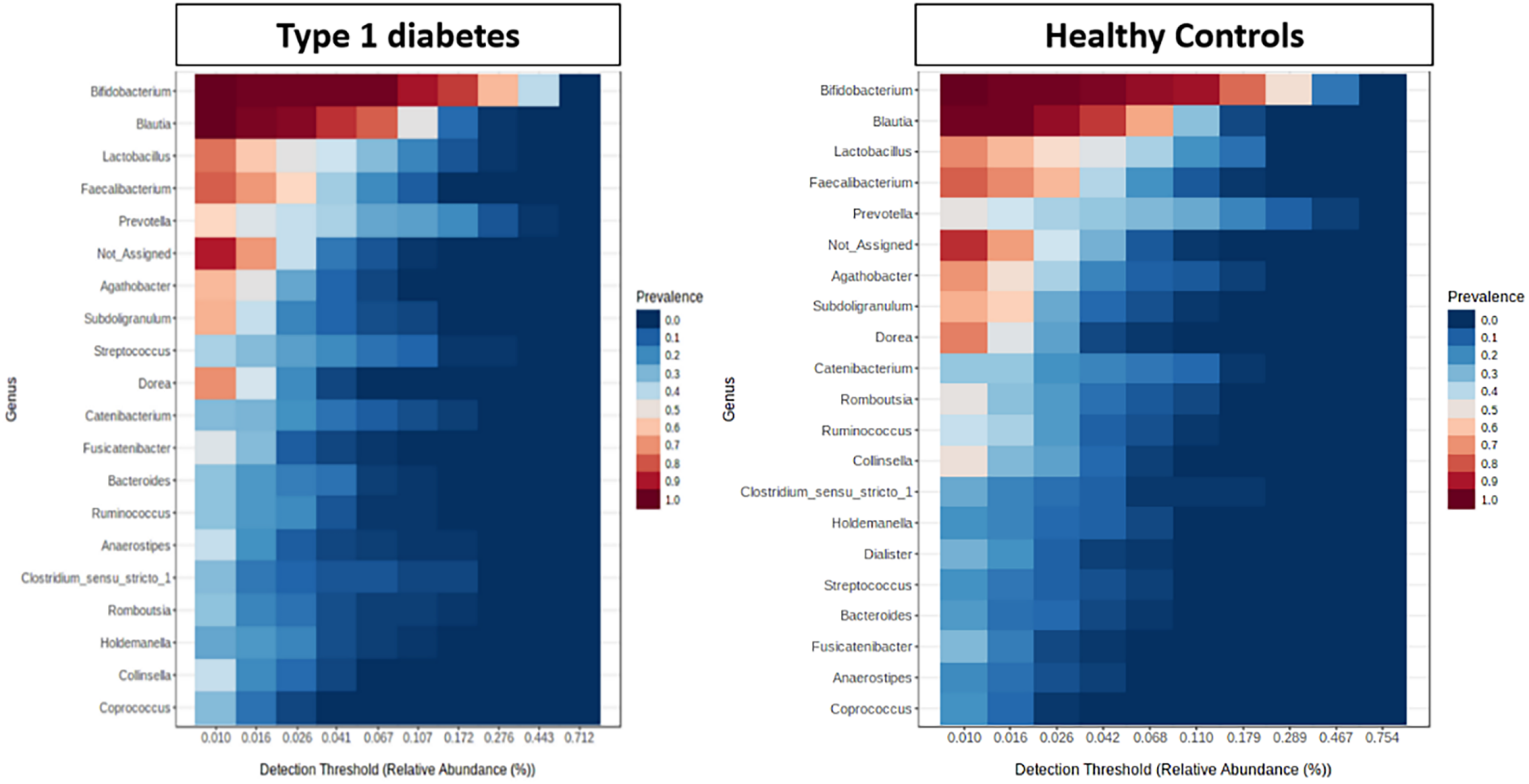
Heat map representing genera of core microbiome in Indian children with type 1 diabetes and healthy age‐, gender‐, and body mass index‐matched controls.

Figure 6 depicts the differences in the prevalence of relative abundance of various microbes in the gut of healthy and the group of children with T1DM. The figure illustrates that the prevalence of *Coprococcus* was low, whereas that of *Bifidobacterium*, *Blautia*, and *Lactobacillus* was high in both groups. *Prevotella*, *Romboustia*, and *Bacteriods* had prevalence between 0.2% and 0.6% on comparing the two groups.

## 4 DISCUSSION

To the best of our knowledge, this is the first study conducted to compare the gut microbiome of Indian children with T1DM and healthy controls. Significant differences were observed in the microbiome at genera level of children with T1DM as compared to the controls, thus indicating a distinct gut microbial signature in children with T1DM. The abundance of SCFAs (butyrate producers) like *Haemophilus* and *Mogibacterium* showed a negative relationship with glycemic control. Finally, SCFAs producers like *Haemophilus* were observed to have a significant association with glycemic control in Indian children with T1DM.

In the present study, no significant differences were observed in the alpha diversity between the two groups. Similar findings have been reported by Leiva‐Gea et al in terms of Chao index (community richness) between T1DM, maturity onset diabetes of the young 2, and healthy control groups.^17^ According to the study conducted by Cinek et al in 2016, there were no differences in alpha diversity (on counting observed taxa [operational taxonomic unit] richness, Chao1, ACE, Shannon, Simpson) and beta diversity (Bray‐Curtis, double principal coordinate analysis [DPCoA]) at the levels of bacterial phyla, classes, or genera.^18^ In a Mexican study performed by Mejia‐Leon et al in 2014, similar findings as our study were reported, stating that the bacterial diversity was similar in both children with T1DM and control groups.^19^

We found that the abundance of Actinobacteria, Firmicutes, and Bacteroidetes was significantly different between the two groups whereas the number of Proteobacteria and Fusobacteria was similar between the groups. These findings were in line with a study carried out by Qin et al.^20^ They concluded that Bacteroidetes and Firmicutes were the most common and dominant phyla reported in their study groups. A similar finding was also reported in a study by Murri et al in children with diabetes, in which the bacterial numbers of Actinobacteria and Firmicutes were significantly decreased whereas that of Bacteroidetes were significantly increased in comparison with healthy children.^21^

In the present study, *Parasutterella* in children with T1DM group was found to have significantly higher abundance as compared to the healthy controls. Similar findings have been demonstrated in the studies conducted by Giongo et al and Qi et al.^22^ An Italian study conducted by Biassoni et al in 2020 on newly diagnosed children with T1DM (age 10 years) concluded that the relative abundance of *Parasutterella* genera was significantly lower in T1DM than healthy controls.^23^ This finding was contradictory to our results, possibly due to the gut microbiome profile during onset of diabetes (as seen in the study by Biassoni et al) being different from that in subjects with long‐standing disease duration (as seen in our study). *Acinetobacter*, *Coriobacteriaceae UCG003*, and *Raoultibacter* were found to be significantly less abundant in children with T1DM than healthy controls in our study. Similar findings were reported in a metagenomic study in 145 European women with diabetes, impaired or normal glucose tolerance. This study reported a depletion of metagenomic clusters assigned to *Coriobacteriaceae* in fecal samples from individuals with diabetes.^24^

SCFAs are essential for the gut microbial community because they play an important role in the regulation of energy balance, inflammatory processes, health, and obesity.^25^ Studies conducted by Brown et al and Goffau et al suggest that low numbers of SCFA (butyrate) producers are related to the late phase of prediabetes, which leads to positivity for multiple autoantibodies. The correlation of certain bacterial findings with the number of positive autoantibodies thus indicates a role of dysbiosis as a regulator of beta‐cell autoimmunity leading to eventual beta‐cell destruction and clinical disease.^7^ In the present study, we found that *Acinetobacter* and *Coriobacteriaceae UCG003*, which are butyrate producers (a type of SCFA) were found to be significantly lower in children with T1DM than in the healthy children. These bacterial differences could be responsible for the altered gut permeability previously described in children with T1DM. A study conducted by Wu et al in 2020 reported that 51 species out of the 118 metagenomic species were altered in the prediabetes and type 2 diabetes groups as compared to those species present in low‐risk normal glucose tolerance. These altered species were found to be potential butyrate producers.^26^ A study conducted by Huang et al in 2020 reported that newly diagnosed patients with T1DM showed an increased proportion of IgA‐bound gut bacteria indicating altered composition of gut microbiome in their stool sample that was negatively associated with the concentrations of SCFAs like acetate.^27^

In our study, the genera like *Butyricicoccus* and *Fournierella* showed a positive association with HbA1c whereas *Haemophilus* and *Mogibacterium* showed a negative association with HbA1c. According to a study conducted by Huang et al, there was a statistically significant negative correlation between HbA1c concentrations and antibody levels of *Haemophilus influenzae type B*.^27^

The strength of our study is that the age‐, gender‐, and BMI‐matched healthy controls enrolled in our study were from the same communities as the participants with T1DM, hence they had similar environmental exposure, lifestyle, and dietary patterns. Thus, we were able to separate the influence of diabetes from other environmental factors contributing to the differences observed in the gut microbiome of the two groups. We could predict the possible abundance of members of the gut microbiome, which may affect HbA1c concentrations in children with diabetes. Our findings may be a useful step in the direction of developing strategies to supplement insulin in better glycemic control in T1DM by modifying the gut microbiota. However, few studies suggest that consumption of dietary fiber crucially affects the composition and diversity of the gut microbiome; we were not able to control the dietary intakes of the study subjects in either group. Further, ours being a cross‐sectional study, we were not able to assess the causal relationship between diabetes control and the gut microbiome. Further, all the study subjects came from a single center and control of diabetes was relatively poor, hence, results may not be applicable to children who are well controlled. Lack of data on pancreatic autoantibodies is another limitation of our study. Finally, our study was also limited by the fact that as no metagenomic sequencing was available, no functional analysis was possible, and the microbiota could be analyzed only down to genera rather than species level.

To conclude, our comparative study of the gut microbiome profile in Indian children with T1DM and healthy controls shows that there are differences in the taxonomial composition of the gut microbiome between the two groups. Our study also suggests that SCFA producers may play an important role in glycemic control. Larger intervention studies are required to assess the causal relationship between diabetes control and the gut microbiome.

## AUTHOR CONTRIBUTIONS

Vaishali Tamahane conceptualized and designed the study, contributed to acquisition of data, and analysis and interpretation of data. Shivang Bhanushali, Nikhil Shah, Abhishek Gupta, Vaman Khadilkar, Ketan Gondhalekar, Anuradha Khadilkar, and Yogesh Shouche conceptualized and contributed to analysis and interpretation of data. All the authors contributed in manuscript writing and checking. All authors have accepted responsibility for the entire content of this submitted manuscript and approved submission.

## CONFLICT OF INTEREST

The authors have no conflict of interest to disclose.

## PERMISSION TO REPRODUCE MATERIAL FROM OTHER SOURCES

The content of this paper is original and no data were reproduced from other sources.

## Data Availability

Data shall be provided on reasonable request.
